# Bis(benzyl­tri­methyl­ammonium) bis­[(4*SR*,12*SR*,18*RS*,26*RS*)-4,18,26-trihy­droxy-12-oxido-13,17-dioxahepta­cyclo­[14.10.0.0^3,14^.0^4,12^.0^6,11^.0^18,26^.0^19,24^]hexa­cosa-1,3(14),6,8,10,15,19,21,23-nona­ene-5,25-dione] sesquihydrate: dimeric structure formation *via* [O—H—O]^−^ *negative charge-assisted hydrogen bonds (–CAHB)* with benzyl­tri­methyl­ammonium counter-ions

**DOI:** 10.1107/S2056989016002899

**Published:** 2016-02-24

**Authors:** Ravell Bengiat, Maayan Gil, Asne Klein, Benny Bogoslavsky, Shmuel Cohen, Joseph Almog

**Affiliations:** a The Institute of Chemistry, The Hebrew University of Jerusalem, Jerusalem, 9190401, Israel

**Keywords:** crystal structure, vasarene, negative charge-assisted hydrogen bond [(−)CAHB], supra­molecular dimer, ninhydrin

## Abstract

The reaction between bis-ninhydrin resorcinol and benzyl­tri­methyl­ammonium fluoride in ethanol has produced the title compound, 2C_10_H_16_N^+^·2C_24_H_13_O_8_^−^·1.5H_2_O, which contains a unique centrosymmetric supra­molecular dimeric entity, where two deprotonated ligands are held together *via* two strong and short [O⋯O = 2.4395 (13) Å] [O—H—O]^−^ bonds of the type *negative charge-assisted hydrogen bonds (–CAHB)*. The central aromatic rings of the ligands create parallel-displaced π–π stacking at an inter­planar distance of 3.381 (1) Å, which helps stabilize the dimer. In the crystal, two symmetry-related solvent water mol­ecules with a site occupancy of 0.75 are attached to the carbonyl groups of the dimer by weaker O—H⋯O hydrogen bonds, forming chains along [101].

## Abstract 2

The title compound forms a supra­molecular dimeric entity *via* [O—H—O]^−^ *negative charge-assisted hydrogen bonds (–CAHB)* following a reaction with benzyl­tri­methyl­ammonium fluoride salt.

## Chemical context   

The vasarene family consists of self-assembled, vase-shaped compounds and their analogues, which are prepared by a one-pot reaction between cyclic vicinal polycarbonyl compounds and polyhy­droxy aromatics (Na *et al.*, 2005 ▸; Almog *et al.*, 2009 ▸). The supra­molecular behaviors of these structures have been an ongoing study in our group, particularly their intriguing feature of selective affinity towards ion-pairs of type *M* ^+^F^−^, *M* being a large monovalent cation (Almog *et al.*, 2012 ▸). A recent study has shown that the multiple oxygen-containing functional groups of these ligands (hemiketals, carbonyls and hydroxyls) play a key role in this supra­molecular binding mechanism by forming dimeric entities *via* strong [O—H—O]^−^ hydrogen-bonding (Bengiat *et al.*, 2016 ▸).

## Structural commentary   

The dimer was formed following the reaction of bis ninhydrin resorcinol (**1**) with benzyl­tri­methyl­ammonium fluoride, in which the fluoride acted as a base removing a proton from the hemiketal hydroxyl group (Scheme). Several factors help in stabilizing this dimeric entity. The first is the π–π stacking of the middle aromatic rings that are parallel-displaced but could almost be considered as a ‘sandwich’ conformation due to the minor angle of displacement (15°). The inter­planar distance between the two rings is also quite short [3.381 (1) Å] supporting the strength of this inter­action (Janiak, 2000 ▸).
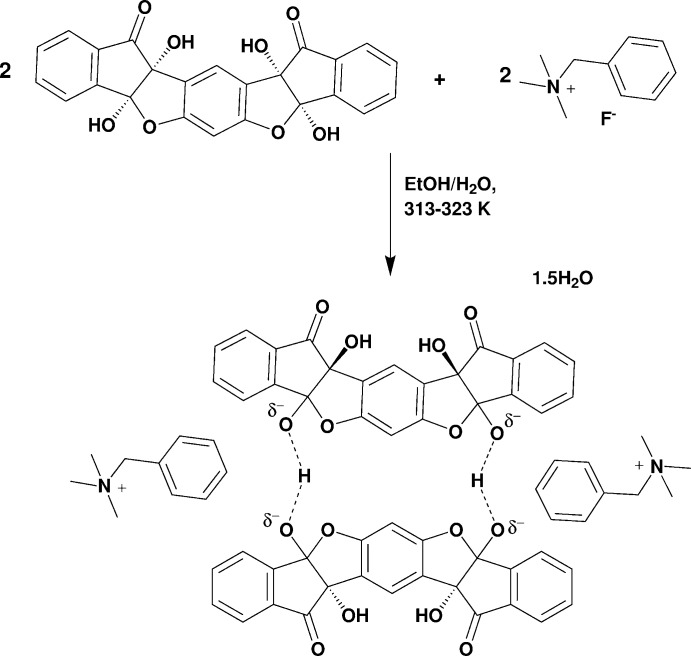


**Figure 1:**
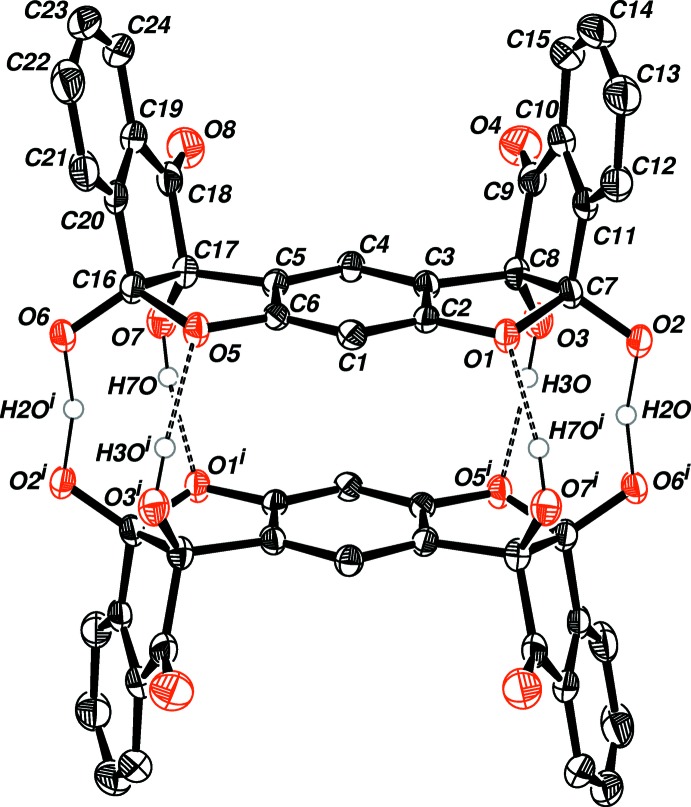
*ORTEP* drawing of the bis ninhydrin resorcinol (**1**) dimer showing 50% probability ellipsoids for non-H atoms. The cations, solvent mol­ecules and aromatic hydrogen atoms have been removed for clarity. [Symmetry code: (i): −*x* + 1, −*y* + 1, −*z* + 1.]

**Figure 2:**
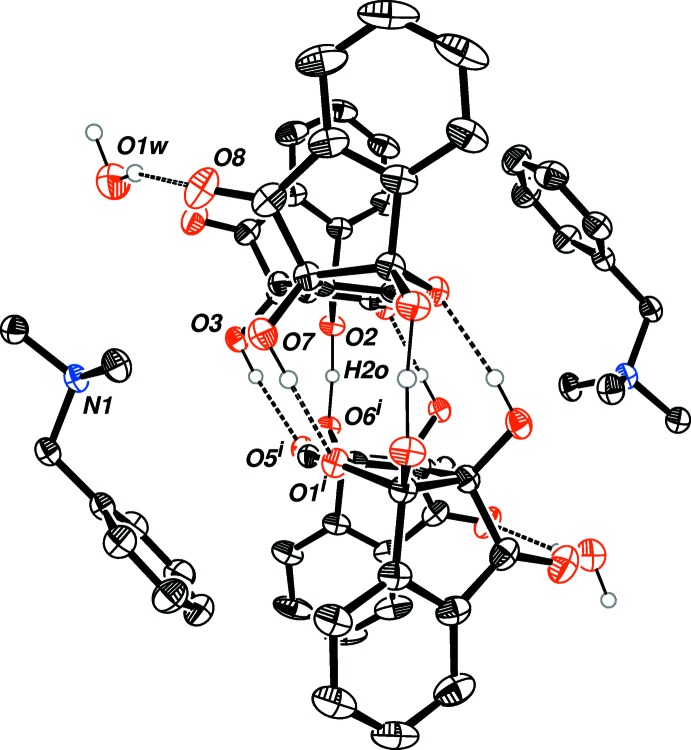
*ORTEP* drawing of the complex showing 50% probability ellipsoids for non-H atoms (side-view). The aromatic and aliphatic hydrogen atoms have been removed for clarity. [Symmetry code: (i): −*x* + 1, −*y* + 1, −*z* + 1.]

**Figure 3:**
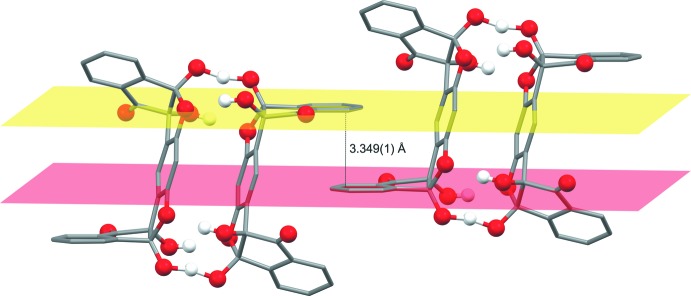
The parallel-displaced π–π stacking between two aromatic rings on the ‘side-walls’ of the ligands of two different cell units showing the inter­planar distance between the rings. The cations, solvent mol­ecules and aromatic hydrogen atoms have been removed for clarity.

 The two [O—H—O]^−^ negative charge-assisted hydrogen bonds (CAHB), although deviating from linearity [164 (2)°], are still considerably strong and short – with an O⋯O distance of 2.4395 (13) Å, corresponding to low-barrier hydrogen bonds (LBHB) (Cleland *et al.*, 1998 ▸). Additional hydrogen bonding (Table 1 ▸) between the remaining hydroxyl groups O7—H7*O*, O3—H3*O* and the etheric hemiketal oxygen atoms O1 and O5, respectively, assist in stabilizing the dimer (Fig. 1 ▸). Fig. 2 ▸ shows that the steric benzyl groups of the cations remain beside the ligands and parallel to each other, with two water mol­ecules hydrogen bonded to the carbonyl groups on the ligands (O1*W*—H2*W*1⋯O8). Two cell units also display parallel-displaced π–π stacking between the aromatic rings of the ‘side-walls’ of the ligands with an inter­planar distance of 3.349 (1) Å (Fig. 3 ▸).

## Database survey   

A survey of the Cambridge Structural Database (Groom & Allen, 2014 ▸) revealed nineteen occurrences of organic compounds containing a similar motif of a negative charge-assisted hydrogen bond (CAHB) of the type [O—H—O]^−^ connecting two carbon atoms. Among them, the shortest O⋯O distances specified range from 2.457 Å (Barczyński *et al.*, 2006 ▸), 2.446 Å (Pan *et al.*, 1996 ▸), 2.437 Å (Polyakova *et al.*, 1983 ▸) to 2.430 Å (Yang *et al.*, 2010 ▸). However, a recent study in our group revealed a much shorter O⋯O distance of 2.404 (3) Å when a completely different dimeric entity was formed in the reaction of (**1**) with tetra­methyl­ammonium fluoride (Bengiat *et al.*, 2016 ▸).

## Synthesis and crystallization   

The ligand (**1**) was prepared by a one-pot synthesis as described in a previously reported procedure (Bengiat *et al.*, 2016 ▸). Bis ninhydrin resorcinol (**1**) (300 mg, 0.7 mmol) was dissolved in hot ethanol (10 mL) and a few drops of water. BnN(Me)_3_F·H_2_O (255 mg, 1.4 mmol) was dissolved in hot ethanol (2 mL). Upon addition of the salt solution to the solution of (**1**), an immediate colour change to intense yellow was observed. A colourless crystalline precipitate was formed after approximately 24 h at RT suitable for single crystal X-ray crystallography.

## Refinement details   

Crystal data, data collection and structure refinement details are summarized in Table 2 ▸. The site occupancy of the water was set at 0.75 during the refinement process, as when defining a value of 1 the *R*-factor increased considerably by 0.7%. Hydroxyl H atoms of the ligand mol­ecules and H atoms of the water mol­ecule were located in a different Fourier map and all H-atom parameters were refined except for those of the water mol­ecule for which only the *U*-parameters were refined. Other H atoms were placed in calculated positions with C—H = 0.93 (aromatic) and 0.96 A (meth­yl), and refined in a riding-model approximation with *U*_iso_(H) = 1.2*U*_eq_(C) for aromatic and aliphatic H atoms and 1.5*U*_eq_(C) for the methyl H atoms.

## Supplementary Material

Crystal structure: contains datablock(s) I, New_Global_Publ_Block. DOI: 10.1107/S2056989016002899/lh5804sup1.cif

Structure factors: contains datablock(s) I. DOI: 10.1107/S2056989016002899/lh5804Isup2.hkl

CCDC reference: 1449570

Additional supporting information:  crystallographic information; 3D view; checkCIF report

## Figures and Tables

**Table 1:** Hydrogen-bond geometry (Å, °)

*D*—H⋯*A*	*D*—H	H⋯*A*	*D*⋯*A*	*D*—H⋯*A*
O1*W*—H2*W*1⋯O8	1.00	1.94	2.898 (2)	160
O1*W*—H1*W*1⋯O4^i^	1.03	2.00	3.028 (2)	174
O7—H7*O*⋯O1^ii^	0.94 (2)	1.85 (2)	2.7818 (14)	171.7 (18)
O3—H3*O*⋯O5^ii^	0.942 (19)	1.942 (19)	2.8796 (14)	173.2 (17)
O2—H2*O*⋯O6^ii^	1.23 (2)	1.23 (2)	2.4395 (13)	164 (2)

Symmetry codes: (i) 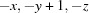; (ii) 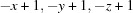.

**Table 2:** Experimental details

Crystal data	
Chemical formula	2C_10_H_16_N^+^·2C_24_H_13_O_8_^−^·1.5H_2_O
*M*_r_	1186.19
Crystal system, space group	Triclinic, *P* 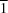
Temperature (K)	173
*a*, *b*, *c* (Å)	10.934 (2), 11.088 (2), 12.402 (2)
α, β, γ (°)	102.873 (3), 106.083 (3), 95.548 (3)
*V* (Å^3^)	1388.0 (4)
*Z*	1
Radiation type	Mo *K*α
μ (mm^−1^)	0.10
Crystal size (mm)	0.31 × 0.19 × 0.15

Data collection	
Diffractometer	Bruker *SMART* CCD
Absorption correction	Multi-scan (*SADABS*; Bruker, 2002 ▸)
*T*_min_, *T*_max_	0.969, 0.985
No. of measured, independent and observed [*I* > 2σ(*I*)] reflections	15809, 5990, 4702
*R*_int_	0.068
(sin θ/λ)_max_ (Å^−1^)	0.639

Refinement	
*R*[*F* ^2^ > 2σ(*F* ^2^)], *wR*(*F* ^2^), *S*	0.045, 0.108, 0.99
No. of reflections	5990
No. of parameters	414
H-atom treatment	H atoms treated by a mixture of independent and constrained refinement
Δρ_max_, Δρ_min_ (e Å^−3^)	0.26, −0.42

Computer programs: *SMART* and *SAINT* (Bruker, 2002 ▸), *SHELXS97*, *SHELXL97* and *SHELXTL* (Sheldrick, 2008 ▸), *ORTEPIII* (Burnett & Johnson, 1996 ▸) and *Mercury* (Macrae *et al.*, 2008 ▸).

## supplementary crystallographic information

### Crystal data

**Table:**

2C_10_H_16_N^+^·2C_24_H_13_O_8_^−^·1.5H_2_O	*Z* = 1
*M**_r_* = 1186.19	*F*(000) = 627
Triclinic, *P*1	*D*_x_ = 1.419 Mg m^−^^3^
Hall symbol: -P 1	Mo *K*α radiation, λ = 0.71073 Å
*a* = 10.934 (2) Å	Cell parameters from 5453 reflections
*b* = 11.088 (2) Å	θ = 2.5–28.0°
*c* = 12.402 (2) Å	µ = 0.10 mm^−^^1^
α = 102.873 (3)°	*T* = 173 K
β = 106.083 (3)°	Prism, colourless
γ = 95.548 (3)°	0.31 × 0.19 × 0.15 mm
*V* = 1388.0 (4) Å^3^	

### Data collection

**Table:**

Bruker SMART CCD diffractometer	5990 independent reflections
Radiation source: fine-focus sealed tube	4702 reflections with *I* > 2σ(*I*)
Graphite monochromator	*R*_int_ = 0.068
φ and ω scans	θ_max_ = 27.0°, θ_min_ = 2.5°
Absorption correction: multi-scan (*SADABS*; Bruker, 2002)	*h* = −13→13
*T*_min_ = 0.969, *T*_max_ = 0.985	*k* = −14→14
15809 measured reflections	*l* = −15→15

### Refinement

**Table:**

Refinement on *F*^2^	Primary atom site location: structure-invariant direct methods
Least-squares matrix: full	Secondary atom site location: difference Fourier map
*R*[*F*^2^ > 2σ(*F*^2^)] = 0.045	Hydrogen site location: inferred from neighbouring sites
*wR*(*F*^2^) = 0.108	H atoms treated by a mixture of independent and constrained refinement
*S* = 0.99	*w* = 1/[σ^2^(*F*_o_^2^) + (0.0623*P*)^2^] where *P* = (*F*_o_^2^ + 2*F*_c_^2^)/3
5990 reflections	(Δ/σ)_max_ < 0.001
414 parameters	Δρ_max_ = 0.26 e Å^−^^3^
0 restraints	Δρ_min_ = −0.42 e Å^−^^3^

### Special details

**Table:**

Geometry. All e.s.d.'s (except the e.s.d. in the dihedral angle between two l.s. planes) are estimated using the full covariance matrix. The cell e.s.d.'s are taken into account individually in the estimation of e.s.d.'s in distances, angles and torsion angles; correlations between e.s.d.'s in cell parameters are only used when they are defined by crystal symmetry. An approximate (isotropic) treatment of cell e.s.d.'s is used for estimating e.s.d.'s involving l.s. planes.
Refinement. Refinement of *F*^2^ against ALL reflections. The weighted *R*-factor *wR* and goodness of fit *S* are based on *F*^2^, conventional *R*-factors *R* are based on *F*, with *F* set to zero for negative *F*^2^. The threshold expression of *F*^2^ > σ(*F*^2^) is used only for calculating *R*-factors(gt) *etc*. and is not relevant to the choice of reflections for refinement. *R*-factors based on *F*^2^ are statistically about twice as large as those based on *F*, and *R*- factors based on ALL data will be even larger.

### Fractional atomic coordinates and isotropic or equivalent isotropic displacement parameters (Å^2^)

**Table:**

	*x*	*y*	*z*	*U*_iso_*/*U*_eq_	Occ. (<1)
C1	0.54556 (12)	0.38574 (12)	0.37869 (11)	0.0210 (3)	
H1	0.6094	0.3351	0.3983	0.025*	
C2	0.41599 (12)	0.34619 (11)	0.36199 (11)	0.0200 (3)	
C3	0.32002 (12)	0.41652 (12)	0.32952 (11)	0.0204 (3)	
C4	0.35280 (12)	0.53425 (12)	0.31428 (11)	0.0214 (3)	
H4	0.2884	0.5830	0.2910	0.026*	
C5	0.48258 (12)	0.57893 (11)	0.33408 (11)	0.0205 (3)	
C6	0.57524 (12)	0.50447 (12)	0.36475 (11)	0.0195 (3)	
C7	0.22477 (12)	0.20971 (12)	0.32385 (11)	0.0226 (3)	
C8	0.18957 (12)	0.34289 (12)	0.31418 (12)	0.0228 (3)	
C9	0.10582 (13)	0.31815 (14)	0.18729 (12)	0.0287 (3)	
C10	0.13239 (13)	0.20071 (13)	0.12196 (12)	0.0293 (3)	
C11	0.19725 (13)	0.13783 (13)	0.19887 (12)	0.0257 (3)	
C12	0.23375 (14)	0.02396 (13)	0.15819 (13)	0.0321 (3)	
H12	0.2773	−0.0201	0.2106	0.039*	
C13	0.20496 (16)	−0.02342 (16)	0.03952 (14)	0.0408 (4)	
H13	0.2289	−0.1013	0.0103	0.049*	
C14	0.14213 (16)	0.03992 (16)	−0.03785 (14)	0.0441 (4)	
H14	0.1257	0.0062	−0.1189	0.053*	
C15	0.10309 (15)	0.15168 (16)	0.00167 (13)	0.0400 (4)	
H15	0.0576	0.1942	−0.0512	0.048*	
C16	0.69013 (13)	0.67546 (12)	0.33598 (12)	0.0231 (3)	
C17	0.54794 (12)	0.70177 (12)	0.32628 (11)	0.0229 (3)	
C18	0.49527 (14)	0.71739 (13)	0.20351 (13)	0.0294 (3)	
C19	0.58105 (14)	0.66924 (13)	0.13771 (12)	0.0314 (3)	
C20	0.68852 (13)	0.63946 (12)	0.21048 (12)	0.0271 (3)	
C21	0.77956 (15)	0.58588 (14)	0.16508 (14)	0.0371 (4)	
H21	0.8517	0.5618	0.2133	0.045*	
C22	0.76163 (19)	0.56874 (17)	0.04711 (15)	0.0517 (5)	
H22	0.8234	0.5336	0.0146	0.062*	
C23	0.6554 (2)	0.60174 (17)	−0.02442 (15)	0.0544 (5)	
H23	0.6465	0.5905	−0.1046	0.065*	
C24	0.56311 (18)	0.65039 (15)	0.01930 (14)	0.0447 (4)	
H24	0.4890	0.6707	−0.0299	0.054*	
C25	0.21050 (13)	0.88120 (12)	0.64347 (12)	0.0254 (3)	
C26	0.32821 (14)	0.96261 (13)	0.69602 (13)	0.0326 (3)	
H26	0.3502	1.0271	0.6621	0.039*	
C27	0.41277 (15)	0.95013 (15)	0.79668 (14)	0.0397 (4)	
H27	0.4925	1.0063	0.8321	0.048*	
C28	0.38232 (16)	0.85660 (15)	0.84619 (14)	0.0411 (4)	
H28	0.4413	0.8480	0.9154	0.049*	
C29	0.26592 (16)	0.77517 (14)	0.79522 (14)	0.0378 (4)	
H29	0.2453	0.7102	0.8291	0.045*	
C30	0.17950 (14)	0.78800 (13)	0.69508 (13)	0.0308 (3)	
H30	0.0988	0.7331	0.6614	0.037*	
C31	0.11721 (13)	0.89919 (13)	0.53629 (12)	0.0259 (3)	
H31A	0.1300	0.9894	0.5384	0.031*	
H31B	0.0283	0.8758	0.5383	0.031*	
C32	0.26207 (13)	0.85536 (14)	0.41394 (14)	0.0315 (3)	
H32A	0.3242	0.8283	0.4737	0.047*	
H32B	0.2839	0.9462	0.4262	0.047*	
H32C	0.2651	0.8121	0.3369	0.047*	
C33	0.09611 (14)	0.68618 (12)	0.40874 (13)	0.0308 (3)	
H33A	0.0940	0.6395	0.3310	0.046*	
H33B	0.0113	0.6678	0.4192	0.046*	
H33C	0.1616	0.6612	0.4675	0.046*	
C34	0.03461 (13)	0.85805 (13)	0.32406 (12)	0.0284 (3)	
H34A	0.0562	0.9477	0.3304	0.043*	
H34B	−0.0530	0.8397	0.3287	0.043*	
H34C	0.0391	0.8090	0.2494	0.043*	
N1	0.12893 (10)	0.82394 (10)	0.42162 (10)	0.0243 (3)	
O1	0.37059 (8)	0.23328 (8)	0.37546 (8)	0.0231 (2)	
O2	0.17312 (9)	0.15028 (8)	0.38748 (8)	0.0260 (2)	
H2O	0.205 (2)	0.203 (2)	0.492 (2)	0.087 (7)*	
O3	0.12328 (9)	0.39817 (9)	0.38979 (9)	0.0269 (2)	
H3O	0.1761 (18)	0.4133 (17)	0.4673 (17)	0.057 (6)*	
O4	0.03367 (10)	0.38624 (11)	0.15004 (10)	0.0428 (3)	
O5	0.69830 (8)	0.55906 (8)	0.38039 (8)	0.0235 (2)	
O6	0.78933 (8)	0.76444 (8)	0.40482 (8)	0.0256 (2)	
O7	0.53759 (9)	0.80979 (8)	0.40608 (9)	0.0266 (2)	
H7O	0.5749 (19)	0.8019 (18)	0.4813 (17)	0.063 (6)*	
O8	0.39802 (10)	0.76058 (10)	0.16867 (10)	0.0417 (3)	
O1W	0.12255 (16)	0.67396 (15)	0.10433 (14)	0.0505 (4)	0.75
H1W1	0.0750	0.6573	0.0168	0.113 (12)*	0.75
H2W1	0.2114	0.7101	0.1091	0.139 (15)*	0.75

### Atomic displacement parameters (Å^2^)

**Table:**

	*U*^11^	*U*^22^	*U*^33^	*U*^12^	*U*^13^	*U*^23^
C1	0.0197 (7)	0.0215 (7)	0.0215 (7)	0.0040 (5)	0.0061 (5)	0.0051 (5)
C2	0.0237 (7)	0.0175 (6)	0.0182 (6)	−0.0004 (5)	0.0071 (5)	0.0041 (5)
C3	0.0181 (6)	0.0213 (7)	0.0205 (7)	−0.0002 (5)	0.0068 (5)	0.0032 (5)
C4	0.0201 (7)	0.0211 (7)	0.0240 (7)	0.0039 (5)	0.0075 (5)	0.0066 (5)
C5	0.0218 (7)	0.0190 (6)	0.0213 (7)	0.0013 (5)	0.0082 (5)	0.0057 (5)
C6	0.0176 (6)	0.0226 (7)	0.0171 (6)	−0.0001 (5)	0.0068 (5)	0.0027 (5)
C7	0.0188 (6)	0.0220 (7)	0.0248 (7)	−0.0023 (5)	0.0063 (5)	0.0043 (5)
C8	0.0192 (7)	0.0229 (7)	0.0269 (7)	0.0008 (5)	0.0093 (6)	0.0063 (6)
C9	0.0196 (7)	0.0353 (8)	0.0316 (8)	−0.0019 (6)	0.0059 (6)	0.0146 (7)
C10	0.0246 (7)	0.0318 (8)	0.0265 (7)	−0.0076 (6)	0.0056 (6)	0.0057 (6)
C11	0.0224 (7)	0.0265 (7)	0.0255 (7)	−0.0064 (5)	0.0091 (6)	0.0038 (6)
C12	0.0343 (8)	0.0291 (8)	0.0317 (8)	−0.0027 (6)	0.0145 (7)	0.0032 (6)
C13	0.0462 (10)	0.0368 (9)	0.0354 (9)	−0.0046 (7)	0.0201 (8)	−0.0035 (7)
C14	0.0488 (10)	0.0472 (10)	0.0258 (8)	−0.0119 (8)	0.0134 (8)	−0.0053 (7)
C15	0.0346 (9)	0.0499 (10)	0.0284 (8)	−0.0099 (7)	0.0036 (7)	0.0111 (7)
C16	0.0232 (7)	0.0213 (7)	0.0254 (7)	−0.0008 (5)	0.0095 (6)	0.0064 (6)
C17	0.0214 (7)	0.0211 (7)	0.0268 (7)	0.0004 (5)	0.0086 (6)	0.0072 (6)
C18	0.0287 (8)	0.0232 (7)	0.0323 (8)	−0.0062 (6)	0.0031 (6)	0.0117 (6)
C19	0.0375 (8)	0.0266 (7)	0.0264 (8)	−0.0099 (6)	0.0083 (6)	0.0080 (6)
C20	0.0292 (7)	0.0229 (7)	0.0268 (7)	−0.0087 (6)	0.0123 (6)	0.0029 (6)
C21	0.0343 (8)	0.0345 (8)	0.0376 (9)	−0.0103 (7)	0.0188 (7)	−0.0034 (7)
C22	0.0559 (12)	0.0501 (11)	0.0416 (10)	−0.0213 (9)	0.0326 (9)	−0.0128 (8)
C23	0.0715 (14)	0.0554 (11)	0.0250 (9)	−0.0299 (10)	0.0201 (9)	−0.0012 (8)
C24	0.0578 (11)	0.0409 (9)	0.0279 (8)	−0.0188 (8)	0.0085 (8)	0.0113 (7)
C25	0.0244 (7)	0.0232 (7)	0.0300 (8)	0.0055 (5)	0.0116 (6)	0.0055 (6)
C26	0.0310 (8)	0.0284 (8)	0.0367 (9)	−0.0012 (6)	0.0090 (7)	0.0095 (6)
C27	0.0307 (8)	0.0411 (9)	0.0403 (9)	−0.0018 (7)	0.0041 (7)	0.0082 (7)
C28	0.0431 (10)	0.0420 (9)	0.0354 (9)	0.0132 (8)	0.0050 (7)	0.0113 (7)
C29	0.0516 (10)	0.0312 (8)	0.0356 (9)	0.0081 (7)	0.0185 (8)	0.0121 (7)
C30	0.0318 (8)	0.0271 (8)	0.0343 (8)	0.0011 (6)	0.0150 (7)	0.0053 (6)
C31	0.0238 (7)	0.0234 (7)	0.0309 (8)	0.0053 (5)	0.0109 (6)	0.0041 (6)
C32	0.0233 (7)	0.0334 (8)	0.0404 (9)	0.0050 (6)	0.0147 (6)	0.0087 (7)
C33	0.0310 (8)	0.0213 (7)	0.0392 (9)	0.0046 (6)	0.0111 (7)	0.0058 (6)
C34	0.0267 (7)	0.0279 (7)	0.0303 (8)	0.0050 (6)	0.0078 (6)	0.0079 (6)
N1	0.0219 (6)	0.0213 (6)	0.0304 (6)	0.0041 (4)	0.0101 (5)	0.0053 (5)
O1	0.0214 (5)	0.0194 (5)	0.0282 (5)	−0.0004 (4)	0.0070 (4)	0.0079 (4)
O2	0.0290 (5)	0.0225 (5)	0.0262 (5)	−0.0042 (4)	0.0116 (4)	0.0057 (4)
O3	0.0209 (5)	0.0288 (5)	0.0330 (6)	0.0037 (4)	0.0123 (4)	0.0070 (4)
O4	0.0339 (6)	0.0509 (7)	0.0441 (7)	0.0119 (5)	0.0050 (5)	0.0203 (6)
O5	0.0175 (5)	0.0244 (5)	0.0298 (5)	0.0005 (4)	0.0083 (4)	0.0092 (4)
O6	0.0217 (5)	0.0266 (5)	0.0252 (5)	−0.0055 (4)	0.0085 (4)	0.0025 (4)
O7	0.0278 (5)	0.0209 (5)	0.0325 (6)	0.0038 (4)	0.0115 (4)	0.0073 (4)
O8	0.0354 (6)	0.0400 (6)	0.0465 (7)	0.0040 (5)	0.0002 (5)	0.0215 (5)
O1W	0.0470 (10)	0.0568 (11)	0.0468 (10)	0.0171 (8)	0.0124 (8)	0.0105 (8)

### Geometric parameters (Å, º)

**Table:**

C1—C6	1.3850 (18)	C20—C21	1.392 (2)
C1—C2	1.3853 (18)	C21—C22	1.387 (2)
C1—H1	0.9500	C21—H21	0.9500
C2—O1	1.3640 (15)	C22—C23	1.388 (3)
C2—C3	1.3912 (18)	C22—H22	0.9500
C3—C4	1.3875 (17)	C23—C24	1.372 (3)
C3—C8	1.5141 (17)	C23—H23	0.9500
C4—C5	1.3915 (18)	C24—H24	0.9500
C4—H4	0.9500	C25—C30	1.3927 (19)
C5—C6	1.3932 (18)	C25—C26	1.3944 (19)
C5—C17	1.5113 (17)	C25—C31	1.501 (2)
C6—O5	1.3638 (15)	C26—C27	1.377 (2)
C7—O2	1.3351 (15)	C26—H26	0.9500
C7—C11	1.5076 (18)	C27—C28	1.376 (2)
C7—O1	1.5164 (16)	C27—H27	0.9500
C7—C8	1.5832 (19)	C28—C29	1.383 (2)
C8—O3	1.4095 (16)	C28—H28	0.9500
C8—C9	1.533 (2)	C29—C30	1.382 (2)
C9—O4	1.2143 (17)	C29—H29	0.9500
C9—C10	1.473 (2)	C30—H30	0.9500
C10—C11	1.384 (2)	C31—N1	1.5258 (17)
C10—C15	1.401 (2)	C31—H31A	0.9900
C11—C12	1.390 (2)	C31—H31B	0.9900
C12—C13	1.381 (2)	C32—N1	1.4977 (17)
C12—H12	0.9500	C32—H32A	0.9800
C13—C14	1.383 (2)	C32—H32B	0.9800
C13—H13	0.9500	C32—H32C	0.9800
C14—C15	1.380 (2)	C33—N1	1.4969 (17)
C14—H14	0.9500	C33—H33A	0.9800
C15—H15	0.9500	C33—H33B	0.9800
C16—O6	1.3354 (15)	C33—H33C	0.9800
C16—C20	1.5129 (19)	C34—N1	1.5028 (17)
C16—O5	1.5129 (16)	C34—H34A	0.9800
C16—C17	1.5863 (19)	C34—H34B	0.9800
C17—O7	1.4095 (16)	C34—H34C	0.9800
C17—C18	1.5268 (19)	O2—H2O	1.23 (2)
C18—O8	1.2176 (18)	O3—H3O	0.942 (19)
C18—C19	1.469 (2)	O7—H7O	0.94 (2)
C19—C20	1.387 (2)	O1W—H1W1	1.0305
C19—C24	1.391 (2)	O1W—H2W1	0.9954
			
C6—C1—C2	115.06 (12)	C19—C20—C21	120.14 (14)
C6—C1—H1	122.5	C19—C20—C16	111.59 (12)
C2—C1—H1	122.5	C21—C20—C16	128.26 (14)
O1—C2—C1	122.70 (11)	C22—C21—C20	117.91 (17)
O1—C2—C3	113.62 (11)	C22—C21—H21	121.0
C1—C2—C3	123.68 (11)	C20—C21—H21	121.0
C4—C3—C2	119.77 (11)	C21—C22—C23	121.45 (17)
C4—C3—C8	130.61 (12)	C21—C22—H22	119.3
C2—C3—C8	109.60 (11)	C23—C22—H22	119.3
C3—C4—C5	118.19 (12)	C24—C23—C22	120.76 (16)
C3—C4—H4	120.9	C24—C23—H23	119.6
C5—C4—H4	120.9	C22—C23—H23	119.6
C4—C5—C6	120.08 (11)	C23—C24—C19	118.16 (17)
C4—C5—C17	130.64 (12)	C23—C24—H24	120.9
C6—C5—C17	109.28 (11)	C19—C24—H24	120.9
O5—C6—C1	122.80 (11)	C30—C25—C26	118.93 (13)
O5—C6—C5	114.02 (11)	C30—C25—C31	121.26 (13)
C1—C6—C5	123.18 (12)	C26—C25—C31	119.74 (12)
O2—C7—C11	115.13 (11)	C27—C26—C25	120.43 (14)
O2—C7—O1	108.79 (10)	C27—C26—H26	119.8
C11—C7—O1	105.41 (10)	C25—C26—H26	119.8
O2—C7—C8	118.43 (11)	C28—C27—C26	120.30 (15)
C11—C7—C8	103.28 (10)	C28—C27—H27	119.8
O1—C7—C8	104.59 (9)	C26—C27—H27	119.8
O3—C8—C3	115.28 (11)	C27—C28—C29	119.98 (15)
O3—C8—C9	110.33 (11)	C27—C28—H28	120.0
C3—C8—C9	108.89 (11)	C29—C28—H28	120.0
O3—C8—C7	116.10 (10)	C30—C29—C28	120.19 (14)
C3—C8—C7	101.74 (10)	C30—C29—H29	119.9
C9—C8—C7	103.55 (11)	C28—C29—H29	119.9
O4—C9—C10	127.80 (14)	C29—C30—C25	120.15 (14)
O4—C9—C8	124.84 (14)	C29—C30—H30	119.9
C10—C9—C8	107.35 (12)	C25—C30—H30	119.9
C11—C10—C15	120.83 (14)	C25—C31—N1	115.06 (11)
C11—C10—C9	109.58 (12)	C25—C31—H31A	108.5
C15—C10—C9	129.59 (14)	N1—C31—H31A	108.5
C10—C11—C12	120.58 (13)	C25—C31—H31B	108.5
C10—C11—C7	112.22 (12)	N1—C31—H31B	108.5
C12—C11—C7	127.15 (13)	H31A—C31—H31B	107.5
C13—C12—C11	118.21 (15)	N1—C32—H32A	109.5
C13—C12—H12	120.9	N1—C32—H32B	109.5
C11—C12—H12	120.9	H32A—C32—H32B	109.5
C12—C13—C14	121.56 (15)	N1—C32—H32C	109.5
C12—C13—H13	119.2	H32A—C32—H32C	109.5
C14—C13—H13	119.2	H32B—C32—H32C	109.5
C15—C14—C13	120.61 (15)	N1—C33—H33A	109.5
C15—C14—H14	119.7	N1—C33—H33B	109.5
C13—C14—H14	119.7	H33A—C33—H33B	109.5
C14—C15—C10	118.19 (15)	N1—C33—H33C	109.5
C14—C15—H15	120.9	H33A—C33—H33C	109.5
C10—C15—H15	120.9	H33B—C33—H33C	109.5
O6—C16—C20	113.58 (11)	N1—C34—H34A	109.5
O6—C16—O5	108.52 (10)	N1—C34—H34B	109.5
C20—C16—O5	107.80 (10)	H34A—C34—H34B	109.5
O6—C16—C17	118.43 (11)	N1—C34—H34C	109.5
C20—C16—C17	103.05 (11)	H34A—C34—H34C	109.5
O5—C16—C17	104.69 (9)	H34B—C34—H34C	109.5
O7—C17—C5	115.40 (11)	C33—N1—C32	110.29 (11)
O7—C17—C18	108.63 (11)	C33—N1—C34	108.61 (10)
C5—C17—C18	109.95 (10)	C32—N1—C34	108.39 (11)
O7—C17—C16	115.99 (11)	C33—N1—C31	110.40 (11)
C5—C17—C16	101.85 (10)	C32—N1—C31	110.87 (10)
C18—C17—C16	104.34 (11)	C34—N1—C31	108.21 (10)
O8—C18—C19	127.60 (14)	C2—O1—C7	107.53 (9)
O8—C18—C17	124.73 (14)	C7—O2—H2O	115.4 (10)
C19—C18—C17	107.66 (12)	C8—O3—H3O	109.4 (12)
C20—C19—C24	121.51 (15)	C6—O5—C16	107.49 (9)
C20—C19—C18	110.42 (12)	C17—O7—H7O	108.1 (12)
C24—C19—C18	128.06 (15)	H1W1—O1W—H2W1	101.6

### Hydrogen-bond geometry (Å, º)

**Table:**

*D*—H···*A*	*D*—H	H···*A*	*D*···*A*	*D*—H···*A*
O1*W*—H2*W*1···O8	1.00	1.94	2.898 (2)	160
O1*W*—H1*W*1···O4^i^	1.03	2.00	3.028 (2)	174
O7—H7*O*···O1^ii^	0.94 (2)	1.85 (2)	2.7818 (14)	171.7 (18)
O3—H3*O*···O5^ii^	0.942 (19)	1.942 (19)	2.8796 (14)	173.2 (17)
O2—H2*O*···O6^ii^	1.23 (2)	1.23 (2)	2.4395 (13)	164 (2)

Symmetry codes: (i) −*x*, −*y*+1, −*z*; (ii) −*x*+1, −*y*+1, −*z*+1.
